# Association between Japanese balanced diet and frailty: the modifying effects of social connections in Japanese older adults

**DOI:** 10.1186/s12877-026-07035-3

**Published:** 2026-01-31

**Authors:** Yaya Li, Hiroko Yoshida, Yuya Akagi, Yuri Tominaga, Mei Nishida, Ayumi Sugibayashi, Liyu Shi, Hanayo Koetaka, Marlon Maus, Gary Yu, Kei Kamide, Michiko Kido, Mai Kabayama

**Affiliations:** 1 Department of Social Epidemiology, Graduate School of Medicine, School of Public Health, Kyoto University, Kyoto, Kyoto Prefecture 606- 8501 Japan; 2 Department of Health Sciences, Graduate School of Medicine, The University of Osaka, Osaka, 565-0871 Japan; 3 Health Research for Action Center, SPH UC Berkeley Investigator, The University of California, Berkeley, USA; 4 Department of Population and Family Health, Mailman School of Public Health, Columbia University, New York, USA

**Keywords:** Dietary patterns, Balanced meal, Kihon checklist, Frailty, Effect modification, Social participation, Sex differences, Older adults, Japan

## Abstract

**Background:**

While the Japanese balanced die is a nutritional-related effort recommended in Japan’s health promotion policies, evidence about its association with frailty is scarce. It’s also worth noting the role of social context, given its wide associations with dietary behaviors and frailty. The present study examined the association between an unbalanced diet and frailty, and whether social connections modify this association in older Japanese adults.

**Methods:**

Data were collected by a cross-sectional survey of 15,302 community-dwelling adults aged ≥ 65 in Osaka, Japan, 2022. Frailty was measured using the validated Kihon Checklist, with a score of ≥ 8 classified as frailty. A balanced meal was defined as a combination of “staple food, main dishes, and vegetables”, in line with Japan’s health promotion policies. Participants without at least one such meal per day were classified as having an “unbalanced diet.” Social connections included social participation, social contact, and social support. Social participation was defined as participation in any of the organizations or groups. Social contact was defined as regular contact with family or friends. Social support was the presence of a supportive social network. Sex-stratified adjusted logistic models were employed to examine the associations between an unbalanced diet and frailty. We further tested interactions between an unbalanced diet and each social connection variable on both multiplicative and additive scales.

**Results:**

The overall prevalence of frailty was 23.1%. Men have a higher proportion of unbalanced diet (29.6%) than women (15.1%). We found an unbalanced diet was associated with frailty in men (OR 1.70, 95% CI 1.38–2.11) and women (OR 1.77, 95% CI 1.38–2.26). In men, significant multiplicative and negative additive interactions were observed between an unbalanced diet and social participation, social contact, and social support (all *p* < 0.05). No significant interactions were found in women.

**Conclusions:**

We identified significant associations between an unbalanced diet and frailty in older Japanese adults. This association varied by social connections status specifically in men. Nutrition-related efforts for frailty may need to consider social context in older men. However, our study was limited by its cross-sectional design.

**Supplementary Information:**

The online version contains supplementary material available at 10.1186/s12877-026-07035-3.

## Background

Healthy aging is a global public health challenge, and preventing frailty is critical to achieving it [1]. Frailty is a predictor of long-term care needs and mortality, but evidence suggests it is a reversible condition with appropriate intervention [2]. Poor nutritional status is a well-established risk factor for frailty and balanced nutrition is one of the strategies to improve nutritional status [3–6]. In Japan, a meal that combines a staple food (carbohydrate such as rice, bread, noodles), main dishes (protein source such as meat, fish, eggs, tofu), and sides (fibers such as vegetables) is defined as a nutritionally balanced “Japanese-style diet”. Promoting this balanced diet is one of the key goals of the national health promotion policies [7–9]. However, evidence examined the association between this Japanese-style balanced diet and frailty is scarce. Only one cross-sectional study reported that a less frequent balanced diet was associated frailty in Japan [10]. However, this previous study failed to capture “frailty” because pre-frail is merged into frailty due to a low prevalence of frailty. This previous study was also limited by a small sample size, and a single item to measure dietary intake. Further research evaluating frailty and the diet-frailty association in a larger sample is needed.

Older adults are especially vulnerable to poor nutrition, and dietary diversity tends to decline with age [11–13]. In Japan, 17.7% of community-dwelling adults aged 65 and older (12.2% of men and 22.4% of women) have been reported to be in poor nutritional status [14]. Simple, culturally familiar dietary guidance may be more effective in this population. The Japanese concept of a “staple food + main dishes + sides” balanced diet is considered an “easy-to-understand” practice; therefore, it may serve as a low-barrier, high-acceptability dietary goal. Understanding the association between this type of balanced diet and frailty may offer insights into sustainable, low-burden strategies to promote healthy aging. Notably, the association between such balanced diet and frailty is likely to be complex. Frailty may limit cooking, grocery shopping, and participation. A review study has reported that the association between poor nutrition and frailty is bidirectional [15].

Social connections may modify the diet-frailty association. Differing frailty and nutritional status were observed in older adults with diverse social connections. For example, the prevalence or risk of frailty is lower among people with active social connections [16–18]; social connections are associated with better nutritional status [19, 20]. According to the convoy model, individuals are surrounded by social connections throughout life course, which has varied structures including social participation, social contact, and social support [21]. In this theorical framework, social connections provide resources and support that influence health and well-being [21]. Thus, it is plausible that social connections may modify the association between dietary behavior and frailty via providing opportunities to access resources and support, or buffering stress level. However, the potential modifying effects remain underexplored.

Gender differences are widely found in frailty, dietary patterns, and social connections [2, 14, 22]. It’s been suggested that associations of social factors with health and dietary status differ between men and women [23, 24]. Gender differences in social connections, such as differences in support sources and type of contact, and gender roles may influence [25]. Therefore, we specify stratified analyses by gender a priori. Understanding sex differences in this Japanese diet-frailty association and the modifying effects of social connections may help inform targeted strategies for prevention. Therefore, the aims of this study are: (1) to examine the association between an unbalanced diet and frailty in older Japanese men and women, and (2) to explore whether social connections—including social participation, social contact, and social support—modify the association between an unbalanced diet and frailty.

## Methods

### Participants and procedures

A postal questionnaire survey was conducted between November and December 2022 among residents aged 65 years and older in three cities in Osaka Prefecture, Japan. Participants were randomly selected through age- and sex-stratified sampling from either the National Health Insurance Registration System or the Late-stage Elderly Medical Care Registration System, both maintained by local government authorities. A total of 15,302 individuals were invited to participate. Participation was voluntary, and all participants provided informed consent by virtue of returning a filled questionnaire. Ethical approval was obtained from the Ethical Review Board of the relevant institution (Approval No. 22243-3).

### Dependent variable: frailty

Frailty was assessed using the Kihon Checklist (KCL), a 25-item screening tool developed by Japan’s Ministry of Health, Labour and Welfare to identify functional decline among community-dwelling older adults [26]. The KCL evaluates multiple domains including basic functioning, psychological and social well-being, oral health, nutrition, physical activity, and cognitive function. The tool’s validity has been previously confirmed [27]. Respondents received one point for each item they indicated difficulty with, for a maximum of 25 points. Incomplete responses were excluded. A total score of 0–3, 4–7, and 8–25 are classified as robust, pre-frail and frail, respectively [27]. We categorized robust and pre-frail individuals into a non-frail group for a binary frailty structure. A score of ≥ 8 is a validated threshold for predicting frailty [27].

### Independent variable: unbalanced diet

Dietary intake was assessed by 24 h dietary recall using a matrix question that captured food consumption across different meals (breakfast, lunch, dinner, and snacks) and food groups (staples [e.g., rice, bread, noodles], main dishes[e.g., meat, fish, eggs, tofu], sides[e.g., vegetables], milk, fruits, and others). The 24-hour period was selected to reduce respondent burden. This dietary assessment tool was adapted from the “Balanced Diet Checksheet” used in local municipal frailty prevention programs. It aligned with the Dietary Guidelines for Japanese and Japan’s nutrition-related health promotion policies [7, 9, 28, 29]. It’s a simple self-report tool designed to capture the practice of the recommended Japanese-style balanced diet [7]. Internal consistency was acceptable (Cronbach’s alpha = 0.67). The assessment is conceptually consistent with the standard question used in the annual National Health and Nutrition Survey, which employs a single-item measure to evaluate the frequency of consuming “meals consisting of a staple food, main dish, and side dish” [30]. We coded whether each meal was a combination of “staple food, main dishes, and sides”, and then we classified those who did not report consuming at least one such combination in breakfast, lunch or dinner as having an “unbalanced diet.” Snacks was not included due to a low prevalence of nutritional balanced snack (0.57%) and this study focuses on main meals. This classification aligns with the national health promotion policies, including the Dietary guidelines for Japanese, the Basic Plan for Food and Nutrition Promotion, and the Health Japan 21 [7–9]. In these policies, the importance of a balanced meal is emphasized.

### Modifying variables: social connections

Social connections included social participation, contact, and support, in line with previous studies [31–33]. Social participation was assessed with the question: “Are you a member of and actively participating in any of the following organizations or groups?” Respondents could select multiple options, including political organizations, local community associations, volunteer or nonprofit groups, civic or consumer cooperatives, religious groups, alumni associations, sports clubs, hobby groups, professional or industry associations, or other organizations. The question was adapted from a national survey on community participation of older adults in Japan [34]. Social participation was defined as engaged in at least one group. Social contact and social support were selected from the social contact and social support items from the frailty check questionnaire suggested in the Project for Prevention of Frailty in the Elderly by Utilizing Dietary Reference Intakes for Japanese [35]. Social contact was assessed by a question “Do you regularly meet with family or friends? (yes / no).” Social support was measured by asking, “When you are not feeling well, do you have someone close by to talk to? (yes / no).”

### Control variables

Potential covariates were determined based on previous research [36, 37] and univariate analysis. Those with *p* < 0.1 in the univariate analysis were included in final multivariate model to control for confounding. Covariates included in the analysis were age (in years), perceived family affluence, marital status, smoking status, and the presence of chronic conditions. Perceived affluence was based on self-reported economic status, with those reporting “very affluent,” “affluent,” or “somewhat affluent” categorized as “affluent,” and others as “not affluent.” Marital status was grouped into “currently married” and “not married.” Smoking status was coded as “current smoker” or “other” (former or never smoker). Chronic conditions included hypertension, diabetes, dyslipidemia, heart disease, depression, and musculoskeletal disorders. Respondents indicated whether they currently had or were being treated for any of these conditions (yes/no). In the sensitivity analysis (Table S3), depression was excluded from the models to avoid potential over-adjustment for constructs embedded in KCL.

### Statistical analysis

Statistical analyses were performed separately for men and women. Demographic characteristics between the frail and non-frail individuals were compared using chi-squared or *t*-tests, where appropriate. We conducted multivariate logistic regression analyses of the associations between an unbalanced diet and frailty, adjusted by covariates illustrated above. To test the modifying effects of social connections, we tested the interaction of an unbalanced det and each social connections variable on both multiplicative and additive scales [38, 39]. We introduced multiplicative interaction terms for an unbalanced diet and each social connections variable (an unbalance diet × social participation, an unbalance diet × social contact, and an unbalance diet × social support) separately. The numbers of individuals within each interaction group for men and women were checked to ensure an adequate sample size. We conducted the likelihood ratio test to access the statistically significance of each interaction. Results confirmed a better fit of interanion effect models (all *p* < 0.05). Additive interactions were assessed by the relative excess risk due to interaction (RERI). To better understand the modifying effects of social connections (social participation, social contact, social support), we visualized these interactions using the marginsplot command in Stata.

Multicollinearity was assessed by variance inflation factors (VIF) test and found no problematic multicollinearity (all VIF < 10, all tolerance > 0.1). Model fit of all the logistic models were assessed using the Hosmer–Lemeshow test, which indicated adequate goodness-of-fit (all *p* > 0.05). Analyses were performed using Stata MP version 19. The significance level less than 0.05 was considered as statistically significant.

## Results

### Participant characteristics

We received 7,503 completed questionnaires, yielding a response rate of 49.0%. Figure S1 shows the inclusion and exclusion criteria. We excluded 132 individuals who were either outside the 65–89 age range or of unknown sex, 246 recipients of long-term care, and 10 institutionalized residents. We further excluding individuals with missing frailty, unbalanced diet, social connections, or covariate information. The final analytic sample included 4,759 participants (33% missing). Proportion of missing data for each variable is shown in Table S1. Comparisons between included and excluded participants are presented in Table S2.

Table 1 presents descriptive statistics for the analytic sample, stratified by sex and frailty status. The mean age was 74.2 years (standard deviation [SD]: 4.9), and approximately half were women (*n* = 2,507; 52.7%). A total of 1,098 participants (23.1%) were classified as frail, with frailty being more prevalent in men than in women (24.8% vs. 21.5%, *p* = 0.007). An unbalanced diet was reported by 29.6% of men and 15.1% of women (*p* < 0.001). Proportion of social participation (61.7% vs. 54.7%, *p* < 0.001), social contact (93.4% vs. 87.8%, *p* < 0.001), and social support (93.1% vs. 89.8%, *p* < 0.001) in women was higher than men. Among both sexes, frail individuals were more likely to have an unbalanced diet, lack social connections, report lower subjective affluence, and have chronic diseases. Among men, frail individuals were also more likely to be current smokers.

**Table 1:** Sex differences in characteristics of frail versus non-frail older adults

	Total (*n* = 4,759)	Men				Women				
		Total (*n* = 2,252)	Frail (*n* = 559)	Non-frail (*n* = 1,693)	*p* value	Total (*n* = 2,507)		Frail (*n* = 539)	Non-frail (*n* = 1,968)	*p* value
Age (year), mean (sd)	74.2 (4.9)	74.5 (4.9)^a^	75.1 (5.1)	74.3 (4.9)	< 0.001	73.9 (4.8)		75.0 (5.0)	73.6 (4.7)	< 0.001
Frail, *n*(%)	1,098 (23.1%)	559 (24.8%)^a^				539 (21.5%)				
Perceived affluence, *n*(%)	2,879 (60.5%)	1,289 (57.2%)^a^	203 (36.3%)	1,086 (64.1%)	< 0.001	1,590 (63.4%)		273 (50.6%)	1,317 (66.9%)	< 0.001
Currently married	3,588 (75.4%)	1,935 (85.9%)^a^	465 (83.2%)	1,470 (86.8%)	0.032	1,653 (65.9%)		339 (62.9%)	1,314 (66.8%)	0.093
Smoking, *n*(%)	398 (8.4%)	313 (13.9%)^a^	109 (19.5%)	204 (12.0%)	< 0.001	85 (3.4%)		25 (4.6%)	60 (3.0%)	0.071
Chronic conditions, *n*(%)	3,053 (64.2%)	1,478 (65.6%)^a^	405 (72.5%)	1,073 (63.4%)	< 0.001	1,575 (62.8%)		392 (72.7%)	1,183 (60.1%)	< 0.001
An unbalanced diet, *n*(%)	1,045 (22.0%)	666 (29.6%)^a^	225 (40.3%)	441 (26.0%)	< 0.001	379 (15.1%)		123 (22.8%)	256 (13.0%)	< 0.001
Social participation, *n*(%)	2,778 (58.4%)	1,232 (54.7%)^a^	216 (38.6%)	1,016 (60.0%)	< 0.001	1,546 (61.7%)		248 (46.0%)	1,298 (66.0%)	< 0.001
Social contact, *n*(%)	4,318 (90.7%)	1,977 (87.8%)^a^	430 (76.9%)	1,547 (91.4%)	< 0.001	2,341 (93.4%)		460 (85.3%)	1,881 (95.6%)	< 0.001
Social support, *n*(%)	4,357 (91.6%)	2,023 (89.8%)^a^	453 (81.0%)	1,570 (92.7%)	< 0.001	2,334 (93.1%)		462 (85.7%)	1,872 (95.1%)	< 0.001

*sd  *standard deviation*,* *p* values were calculated with the *t*-test for numerical differences and chi-squared tests for categorical differences between frail and non-frail individuals

^a^ Statistically significant differences between men and women

### The association between an unbalanced diet and frailty

Table 2 shows the results of the multivariable logistic regression analyses of the main effects models. After adjusting for covariates, an unbalanced diet was associated with higher odds of frailty in both men (odds ratio [OR]: 1.70, 95% confidence interval [CI]: 1.38–2.11) and women (OR: 1.77, 95% CI: 1.38–2.26). The associations remain significant after further adjusting for social participation, social contact, or social support.

**Table 2:** Logistic regression analysis of associations between an unbalanced diet and frailty

	Men				Women			
	Model 1	Model 2	Model 3	Model 4	Model 1	Model 2	Model 3	Model 4
	OR (95% CI)	OR (95% CI)	OR (95% CI)	OR (95% CI)	OR (95% CI)	OR (95% CI)	OR (95% CI)	OR (95% CI)
Unbalanced diet	1.70 (1.38,2.11)	1.65 (1.33,2.04)	1.62 (1.30,2.01)	1.67 (1.34,2.07)	1.77 (1.38,2.26)	1.62 (1.26,2.09)	1.67 (1.30,2.15)	1.68 (1.31,2.16)
Social Participation		0.49 (0.40,0.61)				0.48 (0.39,0.58)		
Social Contact			0.37 (0.28,0.49)				0.28 (0.20,0.40)	
Social Support				0.37 (0.27,0.50)				0.33 (0.23,0.45)
N	2252	2252	2252	2252	2507	2507	2507	2507

*OR* odds ratio, *CI* confidence intervals. Models were adjusted for age, perceived affluence, marriage, smoking, and chronic conditions

### The modifying effects of social participation, social contact, or social support

Table 2 shows that social participation was inversely associated with frailty (men: OR: 0.49, 95% CI: 0.40–0.61; women: OR: 0.48, 95% CI: 0.39–0.58). Social contact was also associated with frailty (men: OR: 0.37, 95% CI: 0.28–0.49; women: OR: 0.28, 95% CI: 0.20–0.40). Social support was negatively associated with frailty (men: OR: 0.37, 95% CI: 0.27–0.50; women: OR: 0.33, 95% CI: 0.23–0.45).

**Fig. 1:**
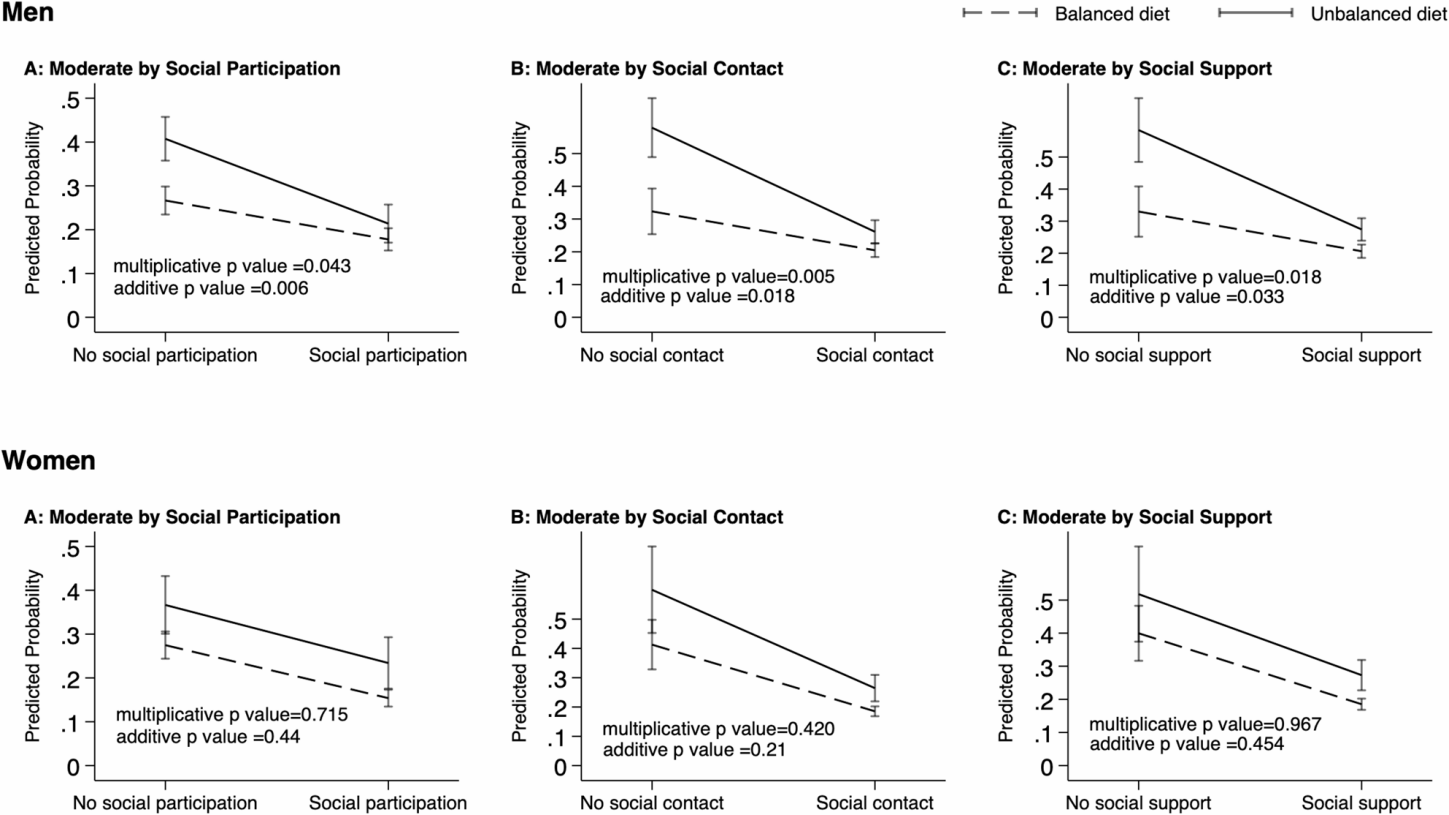
Modifying Effects of Social Connections on the Association between an Unbalanced Diet and Frailty. Note: Predicted probability of frailty and *p* value for interaction of each model was shown. Each model was adjusted by age, perceived affluence, marriage, smoking, and chronic conditions.

Table 3 revealed the interaction models of each interaction items (an unbalance diet × social participation, an unbalance diet × social contact, and an unbalance diet × social support). The results showed significant multiplicative interactions between an unbalanced diet and social connections in men only: an interaction between unbalanced diet and participation on frailty (OR: 0.64, 95% CI: 0.41–0.99, likelihood ratio test *p* value = 0.042), an interaction between unbalanced diet and social contact on frailty (OR: 0.44, 95% CI: 0.25–0.79, likelihood ratio test *p* value = 0.005), an interaction between unbalanced diet and social support on frailty (OR: 0.48, 95% CI: 0.26–0.88, likelihood ratio test *p* value = 0.017). There are also negative additive interactions between an unbalanced diet and social connections (all RERI < 0, all additive *p* value < 0.05) in men. For better visualization, the interaction effects were displayed in Fig. 1.These findings suggest ordinal interactions between an unbalanced diet and each variable of social connections. In other words, the association between an unbalanced diet and frailty was weaker among older men who reported having social participation, social contact, or social support, compared to those who are not. There are no significant multiplicative or additive interactions between an unbalanced diet and social connections in older women (all *p* > 0.05). Sensitivity analyses showed similar results (Table S3).

**Table 3:** Logistic regression models estimating the interaction effects of an unbalanced diet and social connections

	Men			Women		
	Model 1	Model 2	Model 3	Model 1	Model 2	Model 3
	OR (95% CI)	OR (95% CI)	OR (95% CI)	OR (95% CI)	OR (95% CI)	OR (95% CI)
Unbalanced diet	1.99 (1.50,2.65)	3.17 (1.88,5.36)	3.15 (1.78,5.57)	1.56 (1.11,2.18)	2.23 (1.06,4.69)	1.66 (0.82,3.37)
Social Participation	0.58 (0.45,0.74)			0.47 (0.37,0.58)		
Unbalanced diet × Social Participation	0.64 (0.41,0.99)			1.10 (0.67,1.81)		
Social Contact		0.51 (0.35,0.75)			0.31 (0.21,0.45)	
Unbalanced diet × Social Contact		0.44 (0.25,0.79)			0.72 (0.33,1.59)	
Social Support			0.50 (0.33,0.76)			0.32 (0.22,0.48)
Unbalanced diet × Social Support			0.48 (0.26,0.88)			1.02 (0.48,2.16)
N	2252	2252	2252	2507	2507	2507

*OR* odds ratio, *CI* confidence intervals. Models were adjusted for age, perceived affluence, marriage, smoking, and chronic conditions

## Discussions

In this study, we investigated the association between an unbalanced diet and frailty in older Japanese adults, and how social connections influence this association. The proportion of an unbalanced diet was higher among men. We found that an unbalanced diet was associated with frailty in both older men and women; however, the association showed heterogeneity based on social connection status. In older men, social participation, social contact, and social support modified the unbalanced diet-frailty association. In older women, the interactions between an unbalanced diet and social connections were not observed.

Our study supplemented previous research. Previous research has indicated that higher food variety is associated with a reduced risk of frailty in older adults [3, 4, 40]. To our knowledge, only one previous study has reported the association between the Japanese balanced diet and frailty among older Japanese [10]. This previous study found that a lower frequency of eating balanced meals more than twice a day was associated with frailty in both men (OR = 1.74, *n* = 453) and women (OR = 2.30, *n* = 459). Our findings for men are similar, while our reported OR for women (OR = 1.77) is slightly lower. This discrepancy may be due to the difference in sample size and measurement tools. Potential mechanism of the unbalanced diet-frailty association might be related to a lack of nutrients such as vitamins, protein, fibers, calcium, potassium, and others [41–43], which are associated with frailty [43].

Ample evidence supports strong associations between social factors, dietary patterns, and frailty [20, 44]. We extended this literature by jointly examining the modifying effects of social connections in the associations between diet and frailty. Our findings suggest that in Japanese older men, social connections—including social participation, social contact, and social support—modified the unbalanced diet–frailty link. Specifically, in older men with a lack of social connections, an unbalanced diet was more strongly associated with frailty, whereas the association was weaker among those with active social connections. This echoes a recent study which suggested active social connections may “serve as an important protective factor in the relationship between diet and health outcomes” [45]. Potential mechanism remains unclear. One suspected explanation involves stress or inflammation pathways, considering higher inflammatory parameters are associated with frailty [46]. An unbalanced diet maybe proinflammatory due to insufficient intake of vitamins, and trace elements with anti-inflammatory properties [41, 42], while positive social connections have been well documented to be associated with psychosocial buffers such as better mental health, and lower stress levels [47, 48]. A second potential explanation is that social connections provide supportive resources for access to meals and chances for eating with others (meal-sharing), which may help promote energy intake [49]. Sufficient energy intake is reported to prevent frailty [50], which may compensate for the decrease physiological reserves linked with an unbalanced diet. Additionally, eating with others has been proved to improve mental health, which may buffer stress associated with poor nutrition [51]. Moreover, social connections may serve as sources of instrumental support, such as assistance to help recovery from physical deterioration caused by poor nutrition.

However, the potential modifying effects of social connections on the diet-frailty association were only observed in older men but not in the older women. This may be related to the gender differences on social connections and gendered roles. Compared with women, men tend to have smaller and less diversified social networks, participate less in group activities, and received less emotional support [22, 52, 53]. Previous studies have indicated a lower threshold of the effect of social factors on nutrition status, energy intake, and self-reported health among men [23, 24, 54]. Due to the traditional Japanese gender roles, older women might often serve as givers within social connections. For women, especially those with an unbalanced diet, the burden of giving support or others might neutralize the potential buffering benefits of social connections. The possibility of measurement error cannot be excluded, and it might also influence the findings.

This study has several limitations. First, its cross-sectional design precludes conclusions about causal direction, and reverse causality cannot be ruled out. A longitudinal design in the future is needed to evaluate the direction of causality. Second, self-reported data may introduce potential recall bias, common-source bias, or social-desirability bias. The differences between included and excluded participants, although small, also suggest a potential selection bias. Third, the possibility of residual confounding by unmeasured or poorly measured factors cannot be ruled out. The covariate set was predefined but limited by available variables. As a result, the values in our study might be different from the true numbers. Moreover, the study utilized a 24-hour recall measuring dietary practice. We acknowledge that 24-hour recall is limited in its ability to capture long-term dietary practice or variations. We suggest future studies to employ a long-term tracking tool or a longer recall window. In addition, the present study focused on nutritional balance at the meal level. Future studies may consider integrating varied levels to gain a more comprehensive understanding of the associations between this Japanese balanced diet and frailty. Notably, our findings are not intended to negate the importance to consider portion sizes, or the variety of foods consumed. Although the matrix-based “Balanced Diet Checksheet” we adapted aligns with public health policies and previous research, and offers the advantage of minimizing respondent burden, its simplicity limits the availability of precise data on the amount and type of individual nutrients, compared to comprehensive quantitative assessments. We recommend future research to incorporate validated dietary assessment tools such as a Food Frequency Questionnaire. We also suggested using more comprehensive measures of social-network structure and function like the Lubben Network Scale in the future research. Additionally, the KCL measurement overlaps with psychological items. We conducted a sensitive analysis excluding depression from the covariates, and the results remained consistent (Table S3). Furthermore, our findings are based solely on Japanese participants and may not generalize to other populations. Last, all findings should be interpreted considering the potential for type I error due to multiple analyses.

Despite these limitations, the study has notable strengths. It includes a large, community-based sample of older adults. To our knowledge, this is the first study to examine the role of social connections in the diet–frailty pathway in older adults. The Japanese balanced diet has been recommended as a foundation of public health nutrition initiatives. Although we did not use a validated dietary assessment tool such as a Food Frequency Questionnaire, our approach was designed to reflect culturally specific dietary guidelines (“balanced meal”), which may provide practical relevance. Our study complements existing knowledge on the associations between this diet and frailty in older Japanese adults and contributes to public health. Our findings have important implications for practice. For example, the results emphasize a small effort such as having at least intake one balanced meal in a day for community-based nutrition education. Also, the status of social connections should be monitored in community-based nutrition interventions.

## Conclusion

We identified a significant association between an unbalanced diet and frailty among older Japanese adults, and the modifying effects of social connections in Japanese older men. Among older men, social connections modified this association: social participation, social contact, and social support all modified the association between an unbalanced diet and frailty. These findings suggest that nutrition-related strategies to prevent frailty should take into account social context especially among community-dwelling older men. For older women, the present study suggests the importance of the Japanese balanced diet for frailty management. However, the cross-sectional nature of this study limits our ability to infer causal effects. Further longitudinal research is warranted.

## Supplementary Information

Supplementary Material 1.

## Data Availability

Data access is available upon reasonable request, provided that it complies with ethical guidelines.

## Abbreviation

KCL: Kihon Checklist
VIF: variance inflation factor
RERI: relative excess risk due to interaction
