# Supplementary material for: Association between Japanese balanced diet and frailty: the modifying effects of social connections in Japanese older adults
Source: BMC Geriatr. 2026 Jan 31;26:278. doi: 10.1186/s12877-026-07035-3 (PMC12947444; doi:10.1186/s12877-026-07035-3)
Supplement: Supplementary file 1 — Supplementary Material 1. [file 12877_2026_7035_MOESM1_ESM.docx]

Fig. S1. Flow chart of study participants

Table S1. Proportion of missing values by variables

| Variables | Missing values |
| --- | --- |
| Frail, n(%) | 1597 (22.4%) |
| Perceived affluence, n(%) | 178 (2.5%) |
| Currently married | 37 (0.5%) |
| Smoking, n(%) | 78 (1.1%) |
| Chronic conditions, n(%) | 207 (2.9%) |
| An unbalanced diet, n(%) | 66 (0.9%) |
| Social participation, n(%) | 736 (10.3%) |
| Social contact, n(%) | 130 (1.8%) |
| Social support, n(%) | 136 (1.9%) |

*Note.* Variables (Age, Gender) were not included because of no missing values.

Table S2. Differences between excluded and included participants

|  | **Excluded Participants (n = 2,356)** | **Included Participants (n = 4,759)** | **p value** |
| --- | --- | --- | --- |
| Age (year), mean (sd) | 75.8 (5.1) | 74.2 (4.9) | <0.001 |
| Men, n(%) | 1055 (44.8%) | 2252 (47.3%) | 0.043 |
| Frail, n(%) | 225 (9.6%) | 1098 (23.1%) | <0.001 |
| Perceived affluence, n(%) | 1101 (46.7%) | 2879 (60.5%) | <0.001 |
| Currently married | 1714 (72.8%) | 3588 (75.4%) | 0.177 |
| Smoking, n(%) | 220 (9.3%) | 398 (8.4%) | 0.073 |
| Chronic conditions, n(%) | 1404 (59.6%) | 3053 (64.2%) | 0.342 |
| An unbalanced diet, n(%) | 709 (30.1%) | 1045 (22.0%) | <0.001 |
| Social participation, n(%) | 772 (32.8%) | 2778 (58.4%) | <0.001 |
| Social contact, n(%) | 1945 (82.6%) | 4318 (90.7%) | <0.001 |
| Social support, n(%) | 1969 (83.6%) | 4357 (91.6%) | <0.001 |

Note. sd=standard deviation. P-values were calculated with the T-test for numerical differences and chi-squared tests for categorical differences between frail and non-frail individuals.

Table S3. Associations between an unbalanced diet and frailty, and the interaction effects of an unbalanced diet and social connections

|  | **Men** | | |  | **Women** | | |
| --- | --- | --- | --- | --- | --- | --- | --- |
|  | **Odds ratio (95% CI)** | **Odds ratio (95% CI)** | **Odds ratio (95% CI)** |  | **Odds ratio (95% CI)** | **Odds ratio (95% CI)** | **Odds ratio (95% CI)** |
| (a) Main effect models |  |  |  |  |  |  |  |
| Unbalanced diet | 1.65 (1.33,2.04) | 1.61 (1.30,2.00) | 1.66 (1.34,2.06) |  | 1.63 (1.26,2.09) | 1.67 (1.30,2.15) | 1.68 (1.31,2.16) |
| Social Participation | 0.49 (0.40,0.61) |  |  |  | 0.48 (0.39,0.58) |  |  |
| Social Contact |  | 0.37 (0.28,0.49) |  |  |  | 0.28 (0.20,0.40) |  |
| Social Support |  |  | 0.37 (0.27,0.50) |  |  |  | 0.32 (0.23,0.45) |
| (b) Interaction effect models |  |  |  |  |  |  |  |
| Unbalanced diet | 1.99 (1.50,2.65) | 3.17 (1.87,5.35) | 3.14 (1.77,5.56) |  | 1.56 (1.12,2.19) | 2.23 (1.06,4.68) | 1.67 (0.82,3.38) |
| Social Participation | 0.57 (0.45,0.74) |  |  |  | 0.47 (0.37,0.58) |  |  |
| Unbalanced diet × Social Participation | 0.64 (0.41,0.99) |  |  |  | 1.09 (0.66,1.81) |  |  |
| Social Connection |  | 0.51 (0.35,0.75) |  |  |  | 0.31 (0.21,0.45) |  |
| Unbalanced diet × Social Contact |  | 0.44 (0.25,0.79) |  |  |  | 0.72 (0.33,1.59) |  |
| Social Support |  |  | 0.5 (0.33,0.76) |  |  |  | 0.32 (0.22,0.48) |
| Unbalanced diet × Social Support |  |  | 0.48 (0.26,0.88) |  |  |  | 1.01 (0.48,2.16) |
| N | 2252 | 2252 | 2252 |  | 2507 | 2507 | 2507 |

Models were adjusted for age, perceived affluence, marriage, smoking, chronic diseases (depression excluded).
